# Bis(pyridinium) naphthalene-1,5-di­sulfonate dihydrate

**DOI:** 10.1107/S1600536812016960

**Published:** 2012-04-21

**Authors:** Bin Wei

**Affiliations:** a Ordered Matter Science Research Center, Southeast University, Nanjing 211189, People’s Republic of China

## Abstract

The asymmetric unit of the title organic salt, 2C_5_H_6_N^+^·C_10_H_6_O_6_S_2_^2−^·2H_2_O, consists of a pyridinium cation, half a naphthalene-1,5-disulfonate dianion and a water mol­ecule. The dianion has a crystallographically imposed centre of symmetry. In the crystal, N—H⋯O and O—H⋯O hydrogen bonds link cations, anions and water mol­ecules into a three-dimensional network.

## Related literature

For general background to ferroelectric metal-organic frameworks, see: Ye *et al.* (2006 ▶); Zhang *et al.* (2008 ▶, 2010 ▶); Fu *et al.* (2009 ▶).
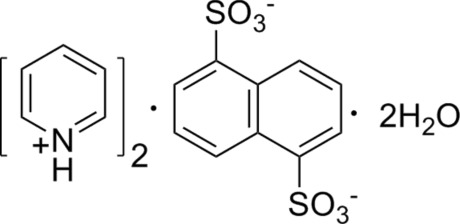


## Experimental

### 

#### Crystal data

2C_5_H_6_N^+^·C_10_H_6_O_6_S_2_^2−^·2H_2_O
*M* *_r_* = 482.52
Monoclinic, 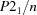

*a* = 9.5876 (19) Å
*b* = 12.065 (2) Å
*c* = 9.843 (2) Å
β = 103.51 (3)°
*V* = 1107.0 (4) Å^3^
*Z* = 2
Mo *K*α radiation
μ = 0.29 mm^−1^
*T* = 293 K
0.55 × 0.44 × 0.36 mm

#### Data collection

Rigaku SCXmini diffractometer
Absorption correction: multi-scan (*CrystalClear*; Rigaku, 2005 ▶) *T*_min_ = 0.860, *T*_max_ = 0.902
11131 measured reflections
2533 independent reflections
2150 reflections with *I* > 2σ(*I*)
*R*_int_ = 0.072

#### Refinement

*R*[*F* ^2^ > 2σ(*F* ^2^)] = 0.056
*wR*(*F* ^2^) = 0.150
*S* = 1.10
2533 reflections
148 parameters
2 restraints
H-atom parameters constrained
Δρ_max_ = 0.52 e Å^−3^
Δρ_min_ = −0.48 e Å^−3^

### 

Data collection: *CrystalClear* (Rigaku, 2005 ▶); cell refinement: *CrystalClear*; data reduction: *CrystalClear*; program(s) used to solve structure: *SHELXTL* (Sheldrick, 2008 ▶); program(s) used to refine structure: *SHELXTL*; molecular graphics: *SHELXTL*; software used to prepare material for publication: *SHELXTL*.

## Supplementary Material

Crystal structure: contains datablock(s) I, global. DOI: 10.1107/S1600536812016960/rz2728sup1.cif

Structure factors: contains datablock(s) I. DOI: 10.1107/S1600536812016960/rz2728Isup2.hkl

Supplementary material file. DOI: 10.1107/S1600536812016960/rz2728Isup3.cml

Additional supplementary materials:  crystallographic information; 3D view; checkCIF report

## Figures and Tables

**Table 1:** Hydrogen-bond geometry (Å, °)

*D*—H⋯*A*	*D*—H	H⋯*A*	*D*⋯*A*	*D*—H⋯*A*
O4—H4*B*⋯O1	0.82	2.07	2.801 (3)	148
O4—H4*A*⋯O2^i^	0.82	1.91	2.731 (3)	174
N1—H10⋯O4^ii^	0.86	1.82	2.667 (3)	170

Symmetry codes: (i) 
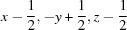
; (ii) 
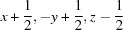
.

**Fig. 1.**
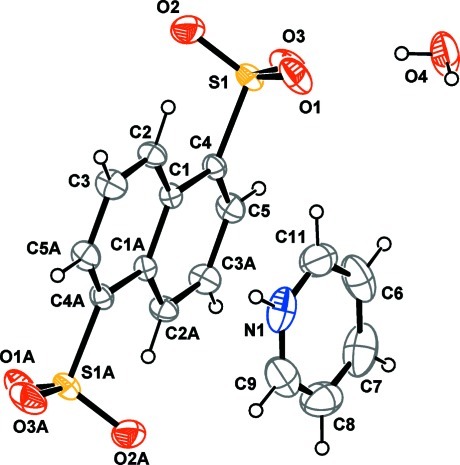
The molecular structure of the title compound, with displacement ellipsoids drawn at the 30% probability level. Atoms labelled with suffix A are generated by the symmetry operator (2-x, -y, 1-z).

**Fig. 2.**
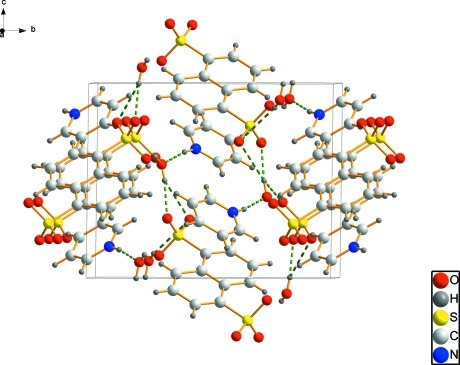
Crystal packing of the title compound viewed along the a axis. Dashed lines indicate hydrogen bonds.

## supplementary crystallographic information

### Comment

Dielectric-ferroelectric constitute an interesting class of materials, comprising organic ligands,metal-organic coordination compounds and organic-inorganic hybrids.(Fu *et al.*, 2009; Zhang *et al.*, 2010; Zhang *et al.*, 2008;Ye *et al.*, 2006). Unfortunately,the dielectric constant of the title compound as a function of temperature indicates that the permittivity is basically temperature-independent, below the melting point (411k-412k) of the compound, we have found that title compound has no dielectric disuniform from 80 K to 405 K. Herein we descibe the crystal structure of this compound.

The asymmetric unit of the title compound (Fig. 1) consists of a pyridinium cation, a half of a naphthalene-1,5-disulfonate anion and a free water molecule, the anion having crystallographically imposed centre of symmetry. The pyridinium and naphthalene rings are oriented to form a dihedral angle of 13.89 (6)°. In the crystal, cations, anions and water molecules are connected by N—H···O and O—H···O intermolecular hydrogen bonds into a three-dimensional structure (Fig. 2; Table 1)

### Experimental

The title compound was obtained by the addition of naphthalene-1,5-disulfonate acid (3.62 g, 0.01 mol) to a solution of pyridine (1.6 g, 0.02 mol) in methanol, in the stoichiometric ratio 1: 2. Good quality single crystals were obtained by slow evaporation of the solvent after six days (yield 52%).

### Refinement

The water H atoms were located in a difference Fourier map and refined as riding, with the O—H distances restrained to 0.82 Å and with *U*_iso_(H) = 1.5*U*_eq_(O). All other H atoms were placed in geometrically idealized positions and constrained to ride on their parent atoms, with C—H = 0.93 Å, N—H = 0.86 Å, and with *U*_iso_(H) = 1.2*U*_eq_(C, N).

### Crystal data

**Table:**

2C_5_H_6_N^+^·C_10_H_6_O_6_S_2_^2^^−^·2H_2_O	*F*(000) = 504
*M**_r_* = 482.52	*D*_x_ = 1.448 Mg m^−^^3^
Monoclinic, *P*2_1_/*n*	Mo *K*α radiation, λ = 0.71073 Å
Hall symbol: -P 2yn	Cell parameters from 3638 reflections
*a* = 9.5876 (19) Å	θ = 3.0–27.5°
*b* = 12.065 (2) Å	µ = 0.29 mm^−^^1^
*c* = 9.843 (2) Å	*T* = 293 K
β = 103.51 (3)°	Block, colourless
*V* = 1107.0 (4) Å^3^	0.55 × 0.44 × 0.36 mm
*Z* = 2	

### Data collection

**Table:**

Rigaku SCXmini diffractometer	2150 reflections with *I* > 2σ(*I*)
Radiation source: fine-focus sealed tube	*R*_int_ = 0.072
Graphite monochromator	θ_max_ = 27.5°, θ_min_ = 3.2°
ω scans	*h* = −12→12
Absorption correction: multi-scan (*CrystalClear*; Rigaku, 2005)	*k* = −15→15
*T*_min_ = 0.860, *T*_max_ = 0.902	*l* = −12→12
11131 measured reflections	2 standard reflections every 150 reflections
2533 independent reflections	

### Refinement

**Table:**

Refinement on *F*^2^	Primary atom site location: structure-invariant direct methods
Least-squares matrix: full	Secondary atom site location: difference Fourier map
*R*[*F*^2^ > 2σ(*F*^2^)] = 0.056	Hydrogen site location: inferred from neighbouring sites
*wR*(*F*^2^) = 0.150	H-atom parameters constrained
*S* = 1.10	*w* = 1/[σ^2^(*F*_o_^2^) + (0.0555*P*)^2^ + 0.5924*P*] where *P* = (*F*_o_^2^ + 2*F*_c_^2^)/3
2533 reflections	(Δ/σ)_max_ < 0.001
148 parameters	Δρ_max_ = 0.52 e Å^−^^3^
2 restraints	Δρ_min_ = −0.48 e Å^−^^3^

### Special details

**Table:**

Geometry. All e.s.d.'s (except the e.s.d. in the dihedral angle between two l.s. planes) are estimated using the full covariance matrix. The cell e.s.d.'s are taken into account individually in the estimation of e.s.d.'s in distances, angles and torsion angles; correlations between e.s.d.'s in cell parameters are only used when they are defined by crystal symmetry. An approximate (isotropic) treatment of cell e.s.d.'s is used for estimating e.s.d.'s involving l.s. planes.
Refinement. Refinement of *F*^2^ against ALL reflections. The weighted *R*-factor *wR* and goodness of fit *S* are based on *F*^2^, conventional *R*-factors *R* are based on *F*, with *F* set to zero for negative *F*^2^. The threshold expression of *F*^2^ > σ(*F*^2^) is used only for calculating *R*-factors(gt) *etc*. and is not relevant to the choice of reflections for refinement. *R*-factors based on *F*^2^ are statistically about twice as large as those based on *F*, and *R*- factors based on ALL data will be even larger.

### Fractional atomic coordinates and isotropic or equivalent isotropic displacement parameters (Å^2^)

**Table:**

	*x*	*y*	*z*	*U*_iso_*/*U*_eq_	
O4	0.4852 (3)	0.2836 (3)	0.5829 (3)	0.1102 (12)	
H4A	0.4806	0.2959	0.4999	0.19 (3)*	
H4B	0.5648	0.2599	0.6216	0.15 (2)*	
S1	0.83962 (6)	0.15020 (5)	0.71768 (6)	0.0461 (2)	
C1	0.9846 (2)	0.04729 (16)	0.53702 (19)	0.0316 (4)	
C2	1.0533 (2)	0.14879 (17)	0.5220 (2)	0.0411 (5)	
H2	1.0350	0.2108	0.5712	0.049*	
C4	0.8853 (2)	0.03502 (17)	0.6239 (2)	0.0348 (4)	
C3	1.1457 (3)	0.15711 (19)	0.4368 (3)	0.0483 (6)	
H3	1.1884	0.2249	0.4269	0.058*	
O3	0.7426 (2)	0.10851 (18)	0.7975 (2)	0.0700 (6)	
C5	0.8229 (3)	−0.0643 (2)	0.6363 (2)	0.0450 (5)	
H5	0.7588	−0.0708	0.6937	0.054*	
O2	0.9728 (2)	0.19099 (18)	0.8046 (2)	0.0659 (6)	
O1	0.7752 (2)	0.23031 (17)	0.6128 (2)	0.0712 (6)	
N1	0.7941 (3)	0.0855 (3)	0.1614 (3)	0.0736 (8)	
H10	0.8605	0.1283	0.1461	0.088*	
C9	0.7696 (4)	−0.0066 (4)	0.0948 (4)	0.0811 (11)	
H9	0.8240	−0.0255	0.0314	0.097*	
C6	0.6178 (5)	0.0513 (4)	0.2786 (4)	0.0933 (13)	
H6	0.5660	0.0718	0.3435	0.112*	
C8	0.6691 (6)	−0.0745 (3)	0.1146 (4)	0.0943 (12)	
H8	0.6521	−0.1410	0.0656	0.113*	
C11	0.7207 (4)	0.1164 (3)	0.2526 (4)	0.0804 (10)	
H11	0.7408	0.1838	0.2989	0.097*	
C7	0.5903 (4)	−0.0464 (4)	0.2074 (6)	0.1002 (15)	
H7	0.5183	−0.0932	0.2223	0.120*	

### Atomic displacement parameters (Å^2^)

**Table:**

	*U*^11^	*U*^22^	*U*^33^	*U*^12^	*U*^13^	*U*^23^
O4	0.0640 (15)	0.206 (4)	0.0579 (14)	0.0563 (18)	0.0094 (11)	−0.0121 (17)
S1	0.0446 (3)	0.0501 (4)	0.0488 (4)	0.0077 (2)	0.0215 (3)	−0.0075 (2)
C1	0.0306 (9)	0.0345 (10)	0.0306 (9)	0.0028 (7)	0.0090 (7)	0.0042 (8)
C2	0.0482 (12)	0.0334 (10)	0.0450 (12)	−0.0010 (9)	0.0177 (10)	−0.0007 (8)
C4	0.0337 (10)	0.0403 (11)	0.0329 (10)	0.0044 (8)	0.0125 (8)	0.0016 (8)
C3	0.0544 (14)	0.0389 (11)	0.0579 (14)	−0.0099 (10)	0.0263 (12)	0.0035 (10)
O3	0.0734 (12)	0.0738 (13)	0.0809 (14)	−0.0020 (11)	0.0546 (11)	−0.0165 (11)
C5	0.0456 (12)	0.0509 (13)	0.0453 (12)	−0.0030 (10)	0.0244 (10)	0.0037 (10)
O2	0.0611 (11)	0.0809 (13)	0.0583 (11)	−0.0078 (10)	0.0192 (9)	−0.0258 (10)
O1	0.0746 (14)	0.0612 (12)	0.0818 (14)	0.0320 (10)	0.0263 (11)	0.0060 (10)
N1	0.0460 (13)	0.094 (2)	0.0767 (18)	−0.0075 (13)	0.0056 (12)	0.0324 (16)
C9	0.087 (2)	0.102 (3)	0.0581 (18)	0.036 (2)	0.0238 (17)	0.0150 (18)
C6	0.080 (2)	0.125 (4)	0.087 (2)	0.039 (2)	0.046 (2)	0.026 (2)
C8	0.116 (3)	0.061 (2)	0.091 (3)	0.006 (2)	−0.005 (3)	−0.0072 (19)
C11	0.090 (3)	0.0665 (19)	0.074 (2)	0.0054 (18)	−0.0035 (19)	−0.0072 (17)
C7	0.063 (2)	0.091 (3)	0.141 (4)	−0.017 (2)	0.012 (2)	0.050 (3)

### Geometric parameters (Å, º)

**Table:**

O4—H4A	0.8207	C5—C3^i^	1.401 (3)
O4—H4B	0.8202	C5—H5	0.9300
S1—O3	1.4413 (19)	N1—C9	1.283 (5)
S1—O1	1.443 (2)	N1—C11	1.317 (5)
S1—O2	1.447 (2)	N1—H10	0.8600
S1—C4	1.779 (2)	C9—C8	1.314 (6)
C1—C2	1.415 (3)	C9—H9	0.9300
C1—C1^i^	1.422 (4)	C6—C11	1.332 (6)
C1—C4	1.428 (3)	C6—C7	1.365 (6)
C2—C3	1.358 (3)	C6—H6	0.9300
C2—H2	0.9300	C8—C7	1.357 (6)
C4—C5	1.358 (3)	C8—H8	0.9300
C3—C5^i^	1.401 (3)	C11—H11	0.9300
C3—H3	0.9300	C7—H7	0.9300
			
H4A—O4—H4B	110.7	C4—C5—H5	119.8
O3—S1—O1	113.62 (13)	C3^i^—C5—H5	119.8
O3—S1—O2	112.87 (13)	C9—N1—C11	122.1 (3)
O1—S1—O2	111.49 (14)	C9—N1—H10	119.0
O3—S1—C4	106.19 (11)	C11—N1—H10	119.0
O1—S1—C4	105.55 (11)	N1—C9—C8	121.1 (4)
O2—S1—C4	106.42 (11)	N1—C9—H9	119.4
C2—C1—C1^i^	118.9 (2)	C8—C9—H9	119.4
C2—C1—C4	122.98 (18)	C11—C6—C7	118.3 (4)
C1^i^—C1—C4	118.1 (2)	C11—C6—H6	120.9
C3—C2—C1	121.0 (2)	C7—C6—H6	120.9
C3—C2—H2	119.5	C9—C8—C7	119.1 (4)
C1—C2—H2	119.5	C9—C8—H8	120.4
C5—C4—C1	120.97 (19)	C7—C8—H8	120.4
C5—C4—S1	118.43 (16)	N1—C11—C6	120.1 (4)
C1—C4—S1	120.60 (16)	N1—C11—H11	120.0
C2—C3—C5^i^	120.5 (2)	C6—C11—H11	120.0
C2—C3—H3	119.8	C8—C7—C6	119.4 (4)
C5^i^—C3—H3	119.8	C8—C7—H7	120.3
C4—C5—C3^i^	120.48 (19)	C6—C7—H7	120.3

Symmetry code: (i) −*x*+2, −*y*, −*z*+1.

### Hydrogen-bond geometry (Å, º)

**Table:**

*D*—H···*A*	*D*—H	H···*A*	*D*···*A*	*D*—H···*A*
O4—H4*B*···O1	0.82	2.07	2.801 (3)	148
O4—H4*A*···O2^ii^	0.82	1.91	2.731 (3)	174
N1—H10···O4^iii^	0.86	1.82	2.667 (3)	170

Symmetry codes: (ii) *x*−1/2, −*y*+1/2, *z*−1/2; (iii) *x*+1/2, −*y*+1/2, *z*−1/2.
